# Interaction of Monocyte-Derived Dendritic Cells with Ara h 2 from Raw and Roasted Peanuts

**DOI:** 10.3390/foods9070863

**Published:** 2020-07-02

**Authors:** Natalija Novak, Soheila J. Maleki, Carmen Cuadrado, Jesus F. Crespo, Beatriz Cabanillas

**Affiliations:** 1 Department of Dermatology and Allergy, University of Bonn Medical Center, DE–53127 Bonn, Germany; Natalija.Novak@ukbonn.de; 2 U.S. Department of Agriculture, Agriculture Research Service, Southern Regional Research Center, New Orleans, LA 70124, USA; soheila.maleki@usda.gov; 3 Department of Food Technology, National Institute of Agricultural, Food Research and Technology (INIA), Ctra. La Coruña Km. 7.5, 28040 Madrid, Spain; cuadrado@inia.es; 4 Department of Allergy, Research Institute Hospital 12 de Octubre, Avenida de Córdoba s/n, 28041 Madrid, Spain; jfcrespo@isciii.es

**Keywords:** Ara h 2, dendritic cells, mannose receptor, monocyte-derived dendritic cells, nuts allergy, peanut allergen, peanut allergy, roasting, thermal processing

## Abstract

Ara h 2 is a relevant peanut allergen linked to severe allergic reactions. The interaction of Ara h 2 with components of the sensitization phase of food allergy (e.g., dendritic cells) has not been investigated, and could be key to understanding the allergenic potential of this allergen. In this study, we aimed to analyze such interactions and the possible mechanism involved. Ara h 2 was purified from two forms of peanut, raw and roasted, and labeled with a fluorescent dye. Human monocyte-derived dendritic cells (MDDCs) were obtained, and experiments of Ara h 2 internalization by MDDCs were carried out. The role of the mannose receptor in the internalization of Ara h 2 from raw and roasted peanuts was also investigated. Results showed that Ara h 2 internalization by MDDCs was both time and dose dependent. Mannose receptors in MDDCs had a greater implication in the internalization of Ara h 2 from roasted peanuts. However, this receptor was also important in the internalization of Ara h 2 from raw peanuts, as opposed to other allergens such as raw Ara h 3.

## 1. Introduction

Peanut allergy is a relevant health issue in many areas of the world [1,2]. Among the allergenic components of peanut, Ara h 2 has been described as a particularly potent allergen. The specific IgE against this allergen seems to be a promising clinical marker for the severity of peanut allergy [3,4,5]. Despite its allergenic potential, Ara h 2 can be modified by thermal treatments in several ways. Some treatments such as boiling are able to potentiate the transfer of Ara h 2 and other related allergens like Ara h 6 and Ara h 7 from the kernel to the cooking water, partially reducing the specific allergenic content of peanuts kernels boiled under specific conditions [6,7,8]. Other treatments, however, can have the opposite effect. Several lines of scientific evidence have shown that roasted and fried peanuts are recognized at a higher level by IgE of peanut-allergic patients than other forms of peanut, such as raw and boiled [9,10,11]. At an individual level, Ara h 2 purified from roasted peanuts was found to have higher IgE-binding properties than Ara h 2 purified from the raw form. In addition to the increase in the allergenic potential of peanut, it has been observed that roasting enhances the trypsin inhibitor activity of Ara h 2 [12]. The chemical reactions that occur in peanuts during roasting, such as Maillard reactions, involving the modification of protein amino groups by reducing sugars during roasting, contribute to the increase of the IgE recognition of roasted peanut allergens [9,13,14]. However, the role of IgE in the recognition of Ara h 2 from raw or roasted peanuts can only partially explain the differences in the allergenic potential of those proteins. IgE production is the consequence of a cascade of immune mechanisms, cells, and molecules involved in the sensitization and effector phases of the allergic response. In that respect, the interaction of Ara h 2 with the components of the immune system involved in the allergic response, specifically in the initial phase named as the sensitization phase, is poorly understood, and its study could be key to understanding the high allergenic potency of specific peanut proteins. In the sensitization phase of allergy, dendritic cells (DCs) have a pivotal role in the modulation of different immune responses, such as allergy or tolerance [15,16]. DCs with a plethora of receptors and mechanisms involved in the recognition and internalization of antigens are the key cells implicated in the higher or lower allergic sensitization to specific proteins [17,18,19,20]. In that sense, previous studies have shown that monocyte-derived dendritic cells (MDDCs) uptake and internalize Ara h 3 from roasted peanuts more efficiently than Ara h 3 purified from raw peanuts, which could explain the higher allergic sensitization potential of roasted Ara h 3 [21]. However, the interaction of DCs with highly relevant peanut allergens such as Ara h 2 and its allergenic sensitization potential has not yet been analyzed. In this study, we aimed to analyze the interaction of Ara h 2 purified from roasted and raw peanuts with human MDDCs, as well as the potential mechanisms involved in this interaction. These analyses could be key to understanding the allergenic impact of Ara h 2 from different forms of peanut.

## 2. Materials and Methods

### 2.1. Ara h 2 Purification from Raw and Roasted Peanuts and Labeling with a Fluorescent Dye

The purification of Ara h 2 from raw and roasted peanuts was carried out by a combination of ammonium sulfate precipitation and ion-exchange chromatography. Raw peanut (*Arachis hypogaea*) flour was defatted with petroleum ether for 6 h. Roasted peanut flour (in defatted form) was generously provided by Golden Peanut, Co (commercial roasted peanuts were chosen in order to use a roasted peanut with the closest characteristics to what consumers normally eat). Defatted flours (10 g) were solubilized in an extraction buffer (500 mL) containing 50 mM Tris-Cl, pH 8.3, 5 mM EDTA, 1 mM PMSF, plus 200 mM NaCl. Ultrasound treatment on ice was used for the homogenization of the solution utilizing a Heat Systems Disrupter at 40% power. Afterwards, the homogenate was centrifugated at 13,000 rpm for 30 min at 4 °C. The precipitation of Ara h 2 from the cleared homogenate was performed by adding a saturated solution of ammonium sulfate while stirring for 30 min on ice. The proteins precipitated by ammonium sulfate were collected after centrifugation at 13,000 rpm for 30 min at 4 °C. The pellet was solubilized in 50 mM Tris, 1 mM EDTA, pH 7; subjected to ion-exchange chromatography for the purification of Ara h 2; and eluted using a salt gradient as previously described [22]. The purities of Ara h 2 from raw and roasted peanuts were evaluated by SDS-PAGE, mass spectrometry, and Western blots using anti-Ara h 2 antibodies and anti-Ara h 1, 3, 6, and 8 antibodies as well as sera IgE from peanut-allergic patients. After these analyses, the purities of Ara h 2 from raw and roasted peanuts were found to be greater than 95%. Ara h 2 purified from raw and roasted peanuts, raw-Ara h 2, and roast-Ara h 2, respectively, were labeled with the fluorescent dye DyLight amine-reactive dye 488 NHS (Thermo Fisher Scientific, Bremen, Germany) as previously described [21]. The raw-Ara h 2 and roast-Ara h 2 labeled with the fluorescent dye were stored protected from light at −20 °C until use.

### 2.2. Generation of Human Monocyte-Derived Dendritic Cells (MDDCs)

The isolation of monocytes was carried out from peripheral blood samples drawn from non-atopic donors after obtaining informed consent (four non-atopic donors; male:female = 1:1; mean age = 34). The study was approved by the Ethics Committee of the University of Bonn. A density gradient protocol utilizing Nycoprep (Progen Biotechnik, Heidelberg, Germany) [23] was used to isolate the monocytes from peripheral blood. Afterwards, monocytes were cultured in VLE-RPMI 1640 (Biochrom, Berlin, Germany) with 10% inactivated fetal calf serum (FCS), 1% antibiotics/antimycotics, 500 U/mL IL-4 (Miltenyi Biotec, Bergisch Gladbach, Germany), and 500 U/mL granulocyte-macrophage colony-stimulating factor (GM-CSF) (Berlex Laboratories, Richmond, VA, USA) for 6 days. Monocytes were fed with half of the above-mentioned cytokine dosages every second day in order to generate MDDCs. Afterwards, MDDCs were washed and resuspended in RPMI 1640 containing 10% inactivated FCS and 1% antibiotics/antimycotics. The cell population was confirmed using CD1a expression as a marker of MDDCs utilizing an anti-human CD1a antibody conjugated with APC and the corresponding isotype control (BD Biosciences, Heidelberg, Germany). Figure S1 in the Supplementary Material shows that high expression of CD1a was obtained.

### 2.3. Internalization Experiments of Raw-Ara h 2 and Roast-Ara h 2 by MDDCs and Effect of Mannose Receptor Blocker

The obtained MDDCs were seeded in flat-bottom, 96-well culture plates (Corning Inc., Corning, NY, USA) at a density of 5 × 10^4^ cells per well. Then, MDDCs were incubated at 37 °C with 10 µg/mL or 50 µg/mL of raw-Ara h 2 or roast-Ara h 2 labeled with the fluorescent dye for 10, 30, and 120 min. After the specific incubation time, MDDCs were collected by gentle centrifugation (300 rpm, 5 min), washed, and subjected to fixation for 20 min with formaldehyde 4% in PBS. Afterwards, the cells were washed, and the internalization of labeled raw-Ara h 2 and roast-Ara h 2 was measured by flow cytometry using the equipment FACS Canto flow cytometer (BD Biosciences, Heidelberg, Germany). The uptake and internalization of raw-Ara h 2 and roast-Ara h 2 were measured as median fluorescence intensity (MFI). In an additional set of experiments, mannan was used as a blocker of the mannose receptor (Sigma-Aldrich, Taufkirchen, Germany) [24,25,26] by adding it to the cell culture plates containing MDDCs at a concentration of 20 µg/mL for 30 min previous to the incubation with raw-Ara h 2 and roast-Ara h 2. The rest of the experiment was carried out as described above. Two technical replicates were used.

### 2.4. Statistical Analysis

The values were expressed as mean ± SEM. The statistical significance was analyzed by Student’s *t*-test. The differences were considered statistically significant with *p*-values lower than 0.05. GraphPad Prism version 8 (GraphPad Software, San Diego, CA, USA) was used for the statistical analyses.

## 3. Results

### 3.1. The Uptake and Internalization of Raw-Ara h 2 and Roast-Ara h 2 by MDDCs Are Time Dependent

**Figure 1:**
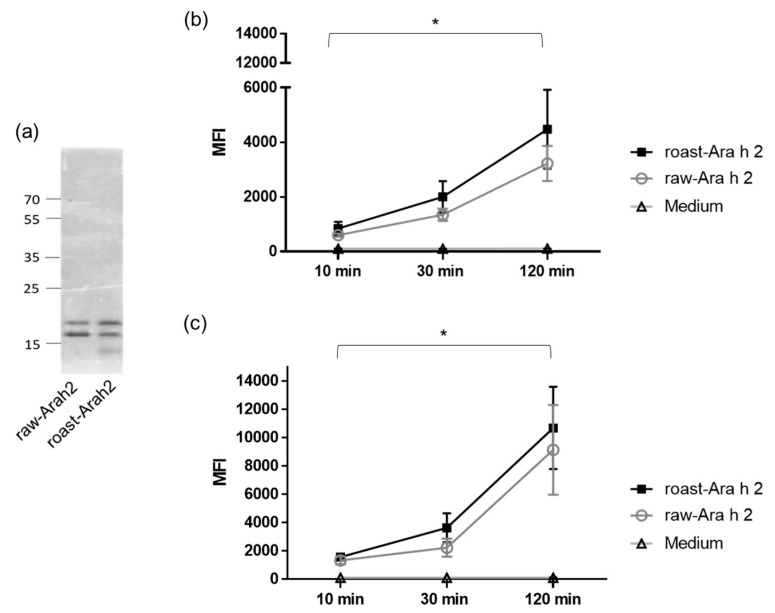
(**a**) SDS-PAGE of purified raw-Ara h 2 and roast-Ara h 2. (**b**,**c**) Internalization of labeled raw-Ara h 2 and roast-Ara h 2 at 10 µg/mL (**b**) and 50 µg/mL (**c**) by monocyte-derived dendritic cells (MDDCs) after 10, 30, and 120 min of allergen incubation, measured by flow cytometry and expressed as median fluorescence intensity (MFI); n = 4 donors. The results are expressed as mean ± SEM. * *p* < 0.05.

In order to study the differences in the uptake and internalization of Ara h 2 from raw and roasted peanuts by MDDCs, Ara h 2 was purified from both forms of peanut and labeled with a fluorescent dye. Figure 1a shows raw-Ara h 2 and roast-Ara h 2 in SDS-PAGE after the purification process and the fluorescent labeling. In the figure, the purified double band of 17 and 21 kDa typical for Ara h 2 can be observed in raw-Ara h 2 and roast-Ara h 2. The faint low-molecular-weight band that can be observed in roasted peanuts could correspond to a minor byproduct derived from roasted peanut purification. However, according to our analyses, the purification of Ara h 2 from raw and roasted peanuts was performed with 95% efficiency. Both labeled allergens were then incubated with human MDDCs for 10, 30, and 120 min at two concentrations (10 and 50 µg/mL), and the internalization of both allergens in those specific conditions were measured by flow cytometry. Figure 1b,c shows that the internalization of both raw-Ara h 2 and roast-Ara h 2 by MDDCs increased in relation to the incubation time at both concentrations used (10 µg/mL (Figure 1b) and 50 µg/mL (Figure 1c)), indicating that the internalization of both allergenic forms was time dependent. Although the internalization of raw-Ara h 2 and roast-Ara h 2 was increased at every time point, the increase was particularly strong and reached statistical significance at 120 min compared with the internalization at 10 min of incubation.

### 3.2. Raw-Ara h 2 and Roast-Ara h 2 Are both Internalized by MDDCs in a Dose-Dependent Manner

**Figure 2:**
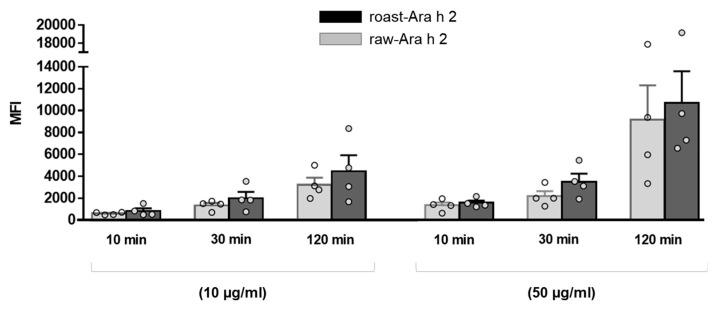
Comparison of the internalization of raw-Ara h 2 (grey bars) and roast-Ara h 2 (black bars) by MDDCs at every time point and at the two concentrations of the allergens used (10 µg/mL and 50 µg/mL). The internalization of the allergens is expressed as MFI; n = 4 donors. The results are represented as mean ± SEM. Individual values are also shown.

The effect of the allergen doses on the internalization of raw-Ara h 2 and roast-Ara h 2 by MDDCs was analyzed and is depicted in Figure 2, which corresponds to the data shown in Figure 1 but represented in dot and bar graphs in order to better visualize the effect of allergen dose and the differences between raw-Ara h 2 and roast-Ara h 2 regarding their internalization. The results showed that, although both allergens were internalized in a dose-dependent manner, the MFI values for roast-Ara h 2 were higher than those for raw-Ara h 2 at every concentration and at every time point, which seems to indicate that roast-Ara h 2 was internalized more efficiently than raw-Ara h 2. Although such differences in the internalization of both allergens did not reach statistical significance, this could be due to the number of samples used (n = 4) (Figure 2).

### 3.3. The Blockage of the Mannose Receptor Decreased the Internalization of Ara h 2 by MDDCs

**Figure 3:**
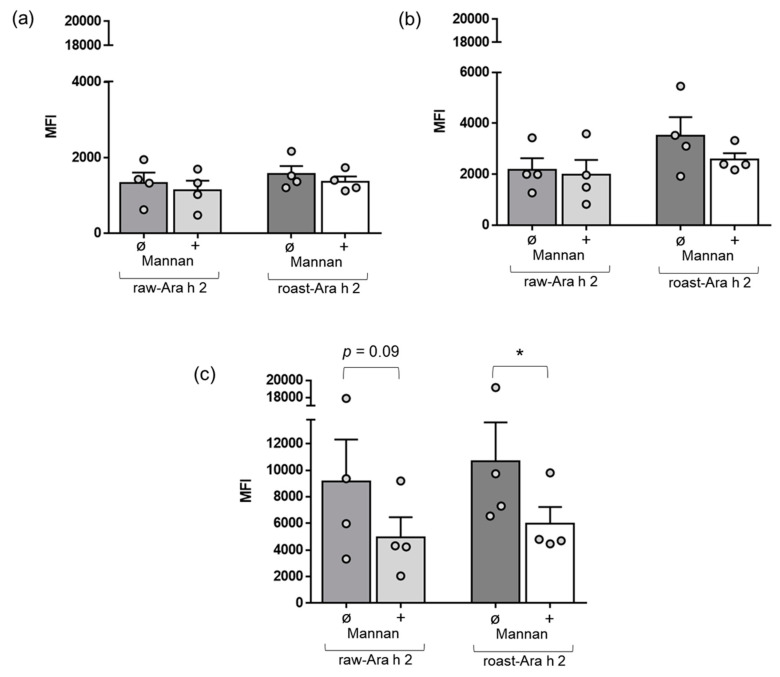
Effect of mannan, a blocker of the receptor, in the internalization blockade of raw-Ara h 2 and roast-Ara h 2 by MDDCs. The graphs show the internalization of raw-Ara h 2 and roast-Ara h 2 by MDDCs at 10 min (**a**), 30 min (**b**), and 120 min (**c**) of allergen incubation with (+) or without (ø) the presence of the blocker mannan. The internalization of the allergens is expressed as MFI; n = 4 donors. The results are represented as mean ± SEM. Individual values are also shown. * *p* < 0.05.

Next, we wanted to characterize the role of a specific receptor of MDDCs, the mannose receptor, in the internalization of raw-Ara h 2 and roast-Ara h 2. In a specific set of experiments, we used mannan, which is a compound that blocks the mannose receptor [24,25,26], and we added it in a preincubation step in the cell cultures of MDDCs before the incubation with the allergens raw-Ara h 2 and roast-Ara h 2. Afterwards, the internalization of both allergens by MDDCs with or without the preincubation step with mannan was measured by flow cytometry as described in the Materials and Methods. Figure 3 shows that mannan did not have remarkable effects on the internalization of either raw-Ara h 2 or roast-Ara h 2 after 10 and 30 min of incubation with the allergens. However, mannan effectively blocked the internalization of raw-Ara h 2 and roast-Ara h 2 when the allergens were incubated with MDDCs for 120 min (Figure 3c). Although mannan strongly contributed to the decrease in the internalization of both allergens by MDDCs at the time point of 120 min, that decrease was significant for roast-Ara h 2 (*p* < 0.05). For raw-Ara h 2, there was an evident decrease in the allergen internalization at 120 min due to the addition of mannan, but the decrease showed a *p*-value of 0.09 (Figure 3c).

## 4. Discussion

DCs have an essential function in the induction of allergy, tolerance, and other immune responses [27,28,29]. However, the role of DCs in the initiation of food allergies and in the differential sensitization potential of certain food proteins is still largely unknown. In that respect, the study of the role of DCs’ interaction with certain food proteins could shed light on the higher or lower allergenic potential of certain food allergens. In the present study, we focused on a food allergen with one of the highest allergenic potentials, Ara h 2, purified from two forms of peanut (raw and roasted), and we analyzed its differential recognition and internalization by human MDDCs. Our results showed that the uptake and internalization of raw-Ara h 2 and roast-Ara h 2 by MDDCs were both time and dose dependent, with a maximum in allergen internalization at 2 h of allergen incubation. Interestingly, although the internalization of roast-Ara h 2 was more efficient at every time point and every concentration than the internalization of raw-Ara h 2, that increase did not reach statistical significance. This could be due to the number of samples used, and a more thorough analysis will be necessary in the future. In our previous study with Ara h 3, we found a statistically significant increase in the internalization of purified Ara h 3 from roasted peanuts compared to Ara h 3 purified from raw peanuts [21]. These apparent differences between Ara h 2 and Ara h 3 might be explained by the different effects that the roasting process has on both allergens. In that sense, previous studies have shown that certain chemical modifications commonly produced during roasting, such as advanced glycation end products (AGEs) and advanced lipoxidation end products (ALEs), were found in Ara h 3 and Ara h 1 but not in Ara h 2 [30,31]. This suggests that roasting impacts Ara h 2 differently than Ara h 3. Differences in structural changes due to roasting may also explain such differences. However, a larger number of samples and thorough analyses will be necessary to confirm these observations.

In order to characterize the role of specific receptors in the uptake and internalization of Ara h 2 by DCs, we focused on the mannose receptor since it has been proven that it can mediate the internalization of different allergens by DCs, contributing to its allergenic sensitization potential [24,25,26]. Our results showed that the mannose receptor seems to have a role to a certain extent in the internalization of roast-Ara h 2 and raw-Ara h 2, since the blockade of such receptor decreased the internalization of both allergens, although in a statistically significant way for roast-Ara h 2. Previous studies also showed that the mannose receptor was involved in the internalization of Ara h 3 purified from roasted peanuts by MDDCs, but in that case, the mannose receptor did not seem to be implicated in the internalization of Ara h 3 from raw peanuts [21]. These results seem to indicate that Ara h 2 purified from roasted and raw peanuts does not have many differences in terms of chemical modifications produced by roasting to those in Ara h 3 since their recognition by the receptor did not show such marked differences as the ones observed for Ara h 3. Previous studies also demonstrated a differential recognition of Ara h 2 and Ara h 3 by certain receptors of DCs. In that context, it has been demonstrated that the receptor of AGE (RAGE) on DCs specifically recognized Ara h 3 but not Ara h 2 due to the presence of AGE modifications in Ara h 3 and not in Ara h 2 [30]. Our study presents limitations such as a low number of donors and that the experiments were carried out without the context of the tissue microenvironment of the skin or the intestine. Our study provides initial experimental insights that will potentially lead to further research exploring the role of mannose (and other) receptors in the internalization mechanisms of peanut allergens by DCs in more physiological tissue environments.

In conclusion, this short communication demonstrates that human MDDCs uptake and internalize Ara h 2 purified from raw and roasted peanuts in a time- and dose-dependent manner. Our study also demonstrates that although the mannose receptor had a greater implication in the internalization of Ara h 2 from roasted peanuts, this receptor is also important in the internalization of Ara h 2 from raw peanuts, as opposed to other allergens such as raw-Ara h 3. Future studies will be necessary in order to analyze the differences in the internalization of peanut allergens by dendritic cells from atopic patients compared to non-atopic individuals.

## Supplementary Materials

The following are available online at https://www.mdpi.com/2304-8158/9/7/863/s1, Figure S1: CD1a expression in MDDCs measured by flow cytometry. A representative experiment is shown.
