# Supplementary material for: Interaction of Monocyte-Derived Dendritic Cells with Ara h 2 from Raw and Roasted Peanuts
Source: Foods. 2020 Jul 2;9(7):863. doi: 10.3390/foods9070863 (PMC7404797; doi:10.3390/foods9070863)
Supplement: Supplementary file 1 [file foods-09-00863-s001.pdf]

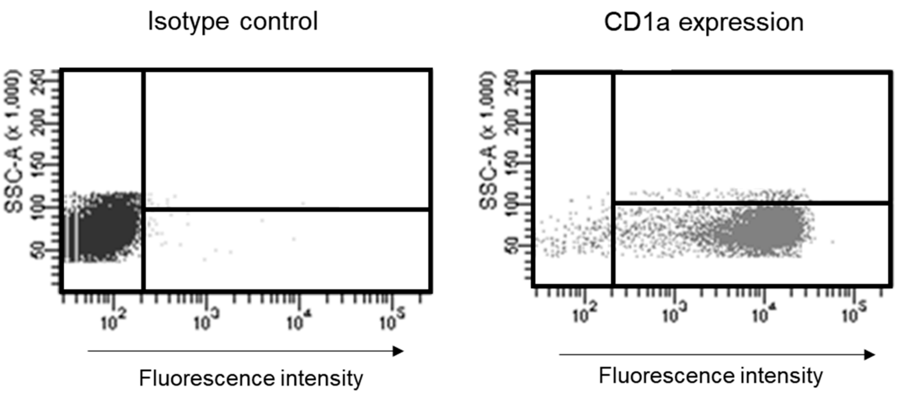

Figure S1. CD1a expression in MDDCs measured by flow cytometry. A representative experiment is shown.
